# Humoral Immune Responses in COVID-19 Patients: A Window on the State of the Art

**DOI:** 10.3389/fimmu.2020.01049

**Published:** 2020-05-15

**Authors:** Gabriel Siracusano, Claudia Pastori, Lucia Lopalco

**Affiliations:** Immunobiology of HIV, Division of Immunology, Transplantation and Infectious Diseases, San Raffaele Scientific Institute, Milan, Italy

**Keywords:** SARS-CoV-2, COVID-19, cytokine storm, antibodies, serological tests

## Abstract

The novel SARS-CoV-2 is a recently emerging virus causing a human pandemic. A great variety of symptoms associated with COVID-19 disease, ranging from mild to severe symptoms, eventually leading to death. Specific SARS-CoV-2 RT-PCR is the standard method to screen symptomatic people; however, asymptomatic subjects and subjects with undetectable viral load escape from the screening, contributing to viral spread. Currently, the lock down imposed by many governments is an important measure to contain the spread, as there is no specific antiviral therapy or a vaccine and the main treatments are supportive. Therefore, there is urgent need to characterize the virus and the viral-mediated responses, in order to develop specific diagnostic and therapeutic tools to prevent viral transmission and efficiently cure COVID-19 patients. Here, we review the current studies on two viral mediated-responses, specifically the cytokine storm occurring in a subset of patients and the antibody response triggered by the infection. Further studies are needed to explore both the dynamics and the mechanisms of the humoral immune response in COVID-19 patients, in order to guide future vaccine design and antibody-based therapies for the management of the disease.

## SARS-CoV-2 Structure, Epidemiology and Clinical Features of the Disease

The severe acute respiratory syndrome corona virus 2 (SARS-CoV-2) (also referred as 2019 novel coronavirus, 2019-nCoV) is the causative agent of a new outbreak emerged in Wuhan City, Hubei province of China, in December 2019, and rapidly spreading all over the world (1–3). Till April 2020, 1,773,084 confirmed cases of novel coronavirus disease 2019 (COVID-19) are documented by the World Health Organization (WHO), with 111,652 deaths globally (4).

SARS-CoV-2 belongs to the beta-coronavirus genus of the *Coronaviridae* family, which includes SARS-CoV, MERS-CoV, bat SARS-related coronaviruses (SARSr-CoV).

Coronaviruses are enveloped, single-stranded positive-sense RNA (+ssRNA) viruses encoding the spike (S), envelope (E), membrane (M), and nucleocapsid (N) structural proteins, 16 non-structural proteins (nsp1–16), and 5–8 accessory proteins (5). The SARS-CoV spike (S) protein is composed of two subunits: the N-terminal S1 subunit contains a receptor-binding domain (RBD) that engages with the angiotensin-converting enzyme 2 (ACE2) receptor on human alveolar epithelial cells of the low respiratory tract. This interaction determines a conformational change in the C-terminal S2 subunit of the S protein that mediates fusion between the viral and host cell membranes. The S protein, particularly its S1 subunit, is highly immunogenic (6). The N protein, abundantly expressed during the infection and highly immunogenic, is involved in the transcription and replication of the RNA and in the packaging of the encapsidated genome into virions (7). The M and E proteins are necessary for virus assembly.

Phylogenetically, SARS-CoV-2 shares 79.6% sequence identity to SARS-CoV and 96% identity to a bat coronavirus, indicating that it may have a zoonotic origin (1, 8).

The majority of Coronaviruses infecting humans are mild, with the exception of SARS-CoV and MERS-CoV, which caused the outbreaks in 2002 and 2012, respectively. The current mortality rate of SARS-CoV-2 is lower than that of SARS-CoV and MERS. However, different from the viruses of the previous outbreaks, SARS-CoV-2 has a higher human-to-human transmission rate. The SARS-CoV-2 S protein binds ACE2 with higher affinity than SARS-CoV, probably leading to the higher transmission across the population (9).

The confirmed transmission modes of SARS-CoV-2 include respiratory droplets and physical contact (10). The first occurs when the mouth and nose mucosae or conjunctiva are exposed to potentially infective respiratory droplets of someone with respiratory symptoms and in close contact (within 1 m). Transmission can occur through contact with contaminated surfaces as well. To date, there have been no reports of fecal–oral transmission of SARS-CoV-2, although a study highlighted that 8 children persistently tested positive on rectal swabs even after nasopharyngeal testing was negative (11). No evidences for intrauterine infection caused by vertical transmission come from the analysis of pregnant women with laboratory-confirmed COVID-19 pneumonia in the late pregnancy and their newborns (12, 13).

Currently, real time reverse transcriptase polymerase reaction (RT-PCR) is the primary diagnostic tool to detect cases of SARS-CoV-2 infection from nasal and pharyngeal swabs and bronchoalveolar lavage (BAL) fluids. In addition, computed tomography imaging and some hematology parameters complement the diagnosis (14).

Typical clinical symptoms of COVID-19 range from asymptomatic state to fever, cough, fatigue and headache, loss of taste and smell, shortness of breath, generalized myalgia, malaise, drowsy, diarrhea, and confusion. Some patients experience more serious illness requiring hospital care, including severe pneumonia symptoms and complications such as acute respiratory distress syndrome (ARDS), which leads to pulmonary edema and lung failure, acute kidney injury, or multiple organ dysfunction and, finally, death. Lymphopenia probably related to lymphocyte apoptosis (15) and interstitial mononuclear inflammatory infiltrates in lung tissues are common clinic-pathological characteristic in COVID-19 patients. Men seem to be at higher risk to develop more severe symptoms as well as subjects suffering from co-morbidities such as cardiovascular and cerebrovascular disease, diabetes and cancer.

## Cytokine Storm in SARS-CoV-2 Infection

Dysregulation of the inflammatory cytokines expression profile was an hallmark during SARS-CoV and MERS-CoV infections and correlated with disease severity and poor prognosis (16, 17).

Several evidences showed that a subgroup of patients with severe COVID-19 experienced an uncontrolled excessive inflammatory response resulting in the cytokine storm syndrome (18–20). A cytokine profile similar to that of secondary haemophagocytic lymphohistiocytosis (sHLH), an under-recognized, hyperinflammatory syndrome characterized by a fulminant and fatal hypercytokinaemia with multiorgan failure, was observed in COVID-19 patients. In addition, elevated ferritin and IL-6 levels observed in 150 confirmed COVID-19 cases suggested that virus-induced hyperinflammation might be one leading cause of fatal outcome (21).

A marked increase of 14 pro- and anti-inflammatory cytokines including IL-1ra (interleukin, IL), IL-2ra, IL-6, IL-10, IL-18, IFN-γ (interferon, IFN), HGF (hepatocyte growth factor), MCP-3 (monocyte chemotactic protein-3), MIG (monokine induced gamma interferon), M-CSF (macrophage colony stimulating factor), G-CSF (granulocyte colony-stimulating factor), MIP-1α (macrophage inflammatory protein 1 alpha) CTACK (cutaneous T-cell-attracting chemokine) and IP-10 (interferon gamma induced protein 10) was found in a cohort of 53 patients with COVID-19 compared to healthy controls. Among them, IP-10, MCP-3 and IL-1ra were significantly associated with disease severity (19), indicating the abnormal inflammatory cytokine release was critical during COVID-19 progression. Indeed, the aberrant expression of cytokines correlated with lung tissue injury and COVID-19 pathogenesis.

Sustained inflammation and cytokine storm in COVID-19 patients were also confirmed at transcriptomic level. The up-regulation of CXCL1, CXCL2, CXCL6, CXCL8, CXCL10/IP-10, CCL2/MCP-1, CCL3/MIP-1A, CCL4/MIP-1B, CCL8, IL33, CCL3L1 was identified in BALF samples, whereas high levels of CXCL10, TNFSF10, TIMP1, C5, IL18, AREG, NRG1, IL-10 were detected in PBMC. The two different gene profiles probably mirrored the differences between the infections in the two cell types. Importantly, increased transcription of the respective chemokines receptors such as CCR2 (CCL2/MCP-1 receptor) and CCR5 (CCL3/MIP-1A receptor) was also observed, indicating the activation of the cytokines-mediated inflammatory signaling pathways (15).

The pro-inflammatory IL-6, normally involved in the regulation of the inflammatory response as well as in B-cell differentiation and consequent antibody production, seems to play a major role in the inflammatory storm. Interestingly, high levels of IL-6 were detected in newborns from COVID-19 mothers (13).

## The Antibody Response Against SARS-CoV-2

### Detection Antibodies and Serological Tests for SARS-CoV-2

The dynamics of the antibody response against SARS-CoV-2 are currently under investigation, as antibodies may be considered potent diagnostic tools to complement RT-PCR based diagnosis.

SARS-CoV-triggered humoral S- and N-specific IgM response reached a peak within 4 weeks and was no more detectable 3 months post symptoms onset (PSO); the switch to IgG often occurred around day 14, and IgGs were detectable up to 36 months (22–24). A summary of the reports analyzing the dynamics of the antibody response during SARS-CoV-2 infection is reported in Table 1.

**Table 1:** Summary of quantitative studies on the antibody dynamics in COVID-19 patients.

**References**	**N. of COVID-19 patients**	**N. of healthy controls**	**IgM**	**IgA**	**IgG**	**Day/week PSO**	**Antigen**	**Test**
Xiao et al. (25)	34	Not reported	322.80 AU/ml^(a)^	Not evaluated	12.40 AU/ml	3 weeks	Not reported	ELISA
			147.92 AU/ml		157.01 AU/ml	4 weeks		
			78.03 AU/ml		163.56 AU/ml	5 weeks		
			21.83 AU/ml		167.16 AU/ml	6 weeks		
Zhao et al. (26)	173	Not reported	82.7%^(b)^	Not evaluated	64,70%	12 days (IgM), 14 days (IgG)	RBD (IgM), NP (IgG)	Double-antigens sandwich (Ab-ELISA), indirect ELISA kit
Jin et al. (27)	43	33	12.1 AU/ml^(a)^	Not evaluated	132.2 AU/ml	Retrospective study, 0–55 days	NP, S	CLIA kits
Guo et al. (28)	82 confirmed, 58 probable	150	400 GMT^(c)^	400 GMT	490.45 GMT	0–7 days	NP	ELISA
			535.8 GMT; *P* = 0.000	597.24 GMT; *P* = 0.000	1325.6 GMT; *P* = 0.000	8-14 days		
			536.31 GMT; *P* = 0.992	723.28 GMT, *P* = 0.156	2690.87 GMT; *P* = 0.000	15–21 days		
			565.69 GMT; *P* = 0.719	831.41 GMT, *P* = 0.538	2974.83 GMT; *P* = 0.72	>21 days		
Szomolanyi-Tsuda and Welsh (29)	214	100	31.8% (NP), 36,4% (S)^(d)^	Not evaluated	31.8% (NP), 40.9% (S)	0–5 days	NP, S	ELISA
			52.6% (NP), 50% (S)		39,5% (NP), 50% (S)	6–10 days		
			72.2% (NP), 83.3% (S)		72.2% (NP), 75.9% (S)	11–15 days		
			81.8% (NP), 96.4% (S)		87.3% (NP), 92.7% (S)	16-20 days		
			81.3% (NP), 87.5% (S)		87.5% (NP), 84.4% (S)	21–30 days		
			83,3% (NP), 100% (S)		100% (NP), 83.3% (S)	31–35 days		
			57.1% (NP), 85.7% (S)		100% (NP), 100% (S)	>35 days		
Liu et al. (30)	58	Not reported	1.72% (IgM only); 94.83 (IgM and IgG)	Not evaluated	3.45% (IgG only); 94.83 (IgM and IgG)	8–33 days	RBD	LFIA
Okba et al. (31)	16	Not reported	81%^(d)^	Not evaluated	100%^(d)^	5 days	NP	ELISA
Zhang et al. (32)	23	93	17% (NP); 26% (RBD)^(b)^	Not evaluated	9% (NP); 43% (RBD)^(b)^	from day 10	NP, RBD	EIA
			88% (NP); 94% (RBD)	Not evaluated	94% (NP); 100% (RBD)	from day 14		

(a) AU/ml, Arbitrary Units/ml;

(b) seroconversion rate (%);

(c) GMT, geometric mean;

(d) *positive rate (%)*.

*ELISA, enzyme-linked immunosorbent assay; CLIA, chemiluminescence immunoassay; LFIA, lateral flow immunoassay; EIA, Enzyme Immuno Assay*.

Xiao et al. showed that all 34 SARS-CoV-2 laboratory confirmed analyzed cases were positive for IgM and IgG at week 3-PSO. IgM levels decreased at week 4; 2 patients were negative at week 5, and additional 2 patients at the end of the observation (week 7). Therefore, in the majority of those patients, the acute phase of infection persisted for more than 1 month. Concomitantly to IgM decrease, IgG levels raised gradually from week 3 to week 7, indicating the activation of the humoral immune response against the virus (25). The authors speculated that the humoral response triggered by SARS-CoV-2 may be similar to that harbored by SARS-CoV.

An additional report on the dynamics of the antibody profile in COVID-19 patients showed that seroconversion appeared sequentially for total antibodies, IgM and IgG, with a median time of 11, 12, and 14 days. Total antibodies were detected by double recombinant antigens sandwich immunoassay (the RBD epitope of the S1 protein and the HRP-conjugated antigen), the IgM μ-chain capture method was used for IgM detection, and indirect ELISA kit based on recombinant NP antigen was used to detect IgG. The seroconversion rate was 93.1, 82.7, 64.7% for total antibodies, IgM and IgG, respectively, and no difference was observed between critical and non-critical patients. Importantly, the sensitivity of antibody detection was lower than the RNA test within 7 days from the onset of the disease (38.3% vs. 66.7%), but raised gradually since day 8 to day 39 PSO, overtaking that of RNA test. More importantly, detectable levels of total antibodies in the sera were found in those patients with undetectable levels of RNA in their respiratory tract samples. This evidence highlighted the extreme importance to combine molecular and serological tests for the accurate diagnosis of COVID-19 patients at different stages of the disease (26). In accordance with this study, Jin et al. reported that the specificity of serum IgM and IgG to detect SARS-CoV-2 infected patients was 90% compared to that of the molecular test. They also registered undetectable levels of any specific antibody up to day 8 PSO in 3 patients (27).

Guo et al. profiled the early antibody response to NP protein in two cohorts of SARS-CoV-2-infected patients. The 90.4% and the 93.3% of 208 patients harbored plasma IgM and IgA, respectively, and the 77.9% of plasma samples were positive for IgG. The median time for IgM and IgA detection was at day 5 PSO (IQR-3-6) and day 14 PSO (IQR 10–18) for IgG (28). The rapid and unexpected IgA seroconversion the authors observed might be an effect of the cytokines storm promoting the germline transcription of both the heavy chain constant α and μ genes. Alternatively, it has been found that T-cell-independent antibody responses stimulate a specialized B cell subset to produce both IgM and IgA during the infection of some pathogens (33). Although the T-cell-independent antibody response against viruses is controversial, some viruses can act *in vivo* as T-cell-independent antigens, eliciting protective, isotype-switched antibodies in the absence of conventional T cell help. Moreover, inactivated virus or virus-like particles can elicit IgM response, but factors induced during active virus infection seem necessary to induce the isotype switch leading to IgG or IgA responses (29).

Liu et al. analyzed a cohort of 214 COVID-19 patients. The 68.2% and the 70.1% of the patients were positive for rN-specific IgM and IgG, respectively; the 77.1% and the 74.3% were positive for rS-specific IgM and IgG, respectively. This data indicated that the detection of rS-specific-IgM was more sensitive compared to that of rN-spcific IgM, probably due to the higher immunogenicity of the S protein compared to that of the N protein. A bioinformatics analysis, indeed, predicted a higher number of B cells epitopes in the S protein than in the NP protein of SARS-CoV-2. In addition, the positive rates of IgM and IgG were low at early stages of the disease (0-10 DPO); conversely, IgM and/or IgG specific for rN and rS reached a peak at 11–15 DPO (30).

The sensitivity of the tests and the epitope on which the test is based on are relevant factors to take into account for the efficient detection of specific SARS-CoV-2 antibodies and timing the humoral response. Therefore, several tests are rapidly developing in many laboratories. Li et al. developed a point-care lateral flow immunoassay (LFIA) test based on the RBD antigen of the SARS-CoV-2 S1 protein that allowed the concomitant detection of IgM and IgG in human blood within 15 min with higher sensitivity than the individual IgG and IgM tests. However, the limit of detection of the test was not determined (34). Importantly, Amanat and collaborators developed sensitive and specific ELISA assays based on the recombinant full-length S protein and RBD epitope allowing the screening and detection of seroconversion upon SARS-CoV-2 infection 3 days PSO (35). Of note, no cross-reactivity from other human coronaviruses was detected, in accordance with another study highlighting that S1 is a specific antigen for SARS-CoV-2 diagnosis as cross-reactive antibodies against the S protein of MERS-CoV were not detected in a COVID-19 patient (31). In addition, strong IgA and IgM responses were uncovered and the IgG3 response was stronger than IgG1 (35).

The sensitivity of the test may pose challenges for the early detection of IgM. Indeed, some patients were more positive for IgG than IgM at the moment of hospitalization and 5 days later; moreover, they had an earlier IgG than IgM seroconversion (32).

SARS-CoV-2 specific antibodies were also detected in 6 sera of infants born from COVID-19 mothers. Five out of six infants and their mothers had high levels of IgG and two of them had high levels of IgM as well. Three out of six infants who had high levels of IgG had normal levels of IgM. However, two of their mothers showed high levels of IgM. How the newborns developed IgM needs further investigations. Indeed, due to their large size, IgM are not usually transferred through the placenta, unless it is affected by some pathology that compromises its structure. The newborn might get contact with the virus if the latter crosses the placenta; however, no virus was detected from RT-PCR analysis (13).

Some studies are investigating the correlation between antigen-specific antibodies and clinical characteristics of COVID-19 patients. Interestingly, patients with comorbidities had lower anti-RBD IgG, but not anti-NP IgM or IgG, than those without comorbidities, although the difference was not significant. No association with age was observed (36).

### Neutralizing Antibodies

Neutralizing antibodies (NAbs) play critical roles in blocking viral infections, thus contributing to viral clearance during acute infection or controlling disease progression during chronic phase. These antibodies are, therefore, useful tools for the protection from viral infection and for the development of effective treatments.

NAbs in the plasma of recovered patients were successfully employed in the passive antibody therapy for SARS-CoV virus- (37), influenza virus- (38) and Ebola virus-infected subjects (39).

The S1 subunit of the S protein, particularly the 193 amino acid length RBD domain (N318-V510), is the main target for antibody-mediated neutralization, probably because it plays major roles during the early stages of infection (40). Studies from SARS-CoV and MERS-CoV demonstrated that many epitopes of the S protein, namely S1-NTD, RBD and S2 are highly immunogenic and can be targets to develop potent NAbs. Several human monoclonal antibodies targeting S1 were developed against SARS-CoV, demonstrating efficient blocking of the binding to the ACE2 receptor in both *in vitro* and animal models. They recognize different epitopes within the S1 subunit, and display different potency of neutralization, alone or in combination (39, 41–43).

Whether SARS-CoV Nabs bind or not SARS-CoV-2 is still controversial. Hoffman et al. demonstrated that the serum from a convalescent SARS-CoV patient neutralized SARS-CoV-2 entry *in vitro* (44). Some studies did not observed any binding (9, 45); however, the SARS-CoV CR3022 NAb bound to SARS-CoV-2 RBD, but it recognized an epitope that did not overlap with the ACE2 binding site within the RBD domain (45). This evidence may suggest a difference in the antigenicity of SARS-CoV and SARS-CoV-2, relevant for the cross-reactivity of NAbs and the design of specific therapeutics against SARS-CoV-2.

**Figure 1:**
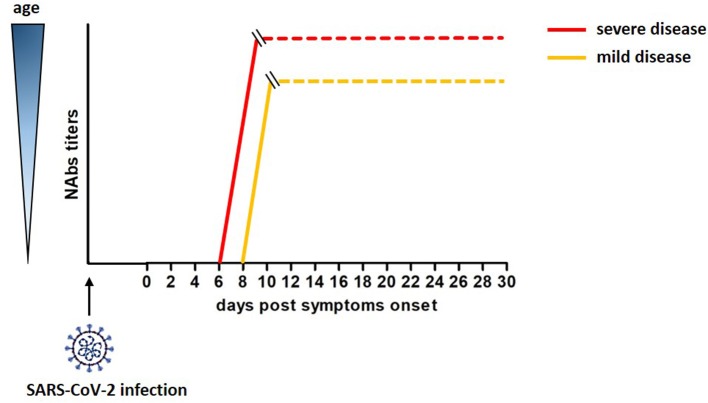
Rough estimate of the development of neutralizing antibodies after SAR-Cov-2 infection and their correlation with age and severity of the disease (46).

A cohort of 175 COVID-19 patients with mild symptoms developed high titers of SARS-CoV-2 specific NAbs targeting the S1, the RBD and the S2 domains of the S protein, with a peak at 10-15 DPO. Interestingly, these NAbs had cross-reactivity but not neutralizing activity against SARS-CoV (46). An unexplored relevant aspect emerged in this study. The titers of SARS-CoV-2 specific NAbs differed across patients and correlated with their age. Elderly and middle age patients displayed higher titers of NAbs than younger patients (46). Of note, Nabs titers in young patients varied, and 10 COVID-19 recovered patients showed Nabs titers below the limit of detection of the assay, although the molecular test confirmed they were SARS-CoV-2-infected. This correlation, recently confirmed by Wang et al. (47), was reported for SARS-CoV and MERS viruses as well; moreover, the strong humoral response observed in aged macaques infected with SARS-CoV related with a severe disease status (48–50). Therefore, age and disease severity may be considered covariates in relation to development of neutralizing antibodies. A rough estimate of the development of neutralizing antibodies after SARS-CoV-2 infection is reported in Figure 1.

No studies on the duration of SARS-CoV-2 specific NAbs have been reported so far. In a cohort study of 56 SARS-CoV convalescent patients, specific IgG and neutralizing antibodies were highly correlated, and persisted for 24 months, despite the decline of their titers (51). Another study showed the 74.2% and the 83.9% of the patients were positive for IgG and neutralizing antibodies 36 months PSO (24). An observational cohort study including 16 COVID-19 patients whose serum samples were collected 14 days PSO showed that the majority of patients harbored neutralizing IgM and IgG against both NP and RBD (36). NP is highly immunogenic, although smaller than S, lacks of glycosylation sites, and induces antibodies earlier than S during the infection, thus contributing to neutralization; therefore, anti-NP-specific antibodies might play a key role during the early stages of acute infection (52).

Discovering the epitopes enabling to elicit humoral responses against SARS-CoV-2 is relevant for the development of specific monoclonal antibodies for therapy or prophylaxis.

A bioinformatics analysis through the Immune Epitope Database and Analysis Resource (IEDB) revealed that the S protein had the highest number of predicted B cell epitopes, particularly the regions 491–505, 558–562, 703–704, 793–794, 810, 914, and 1,140–1,146; however, besides the S protein, the M protein and NP phospoprotein contained B cell immunodominat regions as well (53). Interestingly, the sequences of the B cell immunodominat regions of SARS-CoV were conserved in SARS-CoV-2. Of note, convalescent SARS-CoV patients harbored NAbs directed against the epitopes of five of these regions (54, 55). Moreover, two regions (1–25 and 131–152) within the M protein triggered high IgM and IgG responses (56, 57); the 156–175 region within the NP protein was reactive against sera from SARS patients and showed immunogenicity in a broad spectrum of species, including humans (58).

Plasma of convalescent COVID-19 patients were used to treat 10 severe cases of SARS-CoV-2 infections. The dose (200 mL) of plasma was well tolerated and viremia decreased in 7 days with the concomitant improvement of the clinical symptoms within 3 days (59). This data strongly suggests that the deeper characterization of plasma from recovered patients might give important information for the development of effective antibody-based therapies to treat COVID-19 patients.

### Antibody-Dependent Enhancement (ADE)

An opening question rely on the huge difference in the severity of COVID-19 ranging from asymptomatic, low, mild and severe cases.

Tetro speculated that subjects who experienced the most severe forms of the disease might have been primed by one or more coronavirus exposure leading to the effects of Antibody Dependent Enhancement (ADE) of the SARS-CoV-2 infection (60). The antibodies elicited by a previous contact with a virus might not completely neutralize a second infection and, conversely, form complexes with the second virus or virus-activated complement components that interact with the Fc or complement receptors on susceptible cells, thereby facilitating viral entry (61, 62). In addition, ADE modulates the immune response, triggering inflammation, cytokine storm ad lymphopenia (60), responsible for the poor outcome of the disease.

ADE has been extensively investigated in dengue virus (63–65), and observed in HIV (66) and Ebola (62) infections as well. With respect to coronaviruses, antibodies induced by the SARS-CoV S protein enhanced the viral entry into the cells expressing the Fc receptor (67–69). Liu et al. showed that during acute SARS-CoV infection, anti–S-IgG altered macrophages functions by abrogating their wound-healing response, partially through FcγRs. Concomitantly, anti–S-IgG decreased TGF-β production, while inducing pro-inflammatory cytokine IL-8 and MCP1 production and inflammatory macrophage accumulation in the lung, finally leading to acute lung injury (70). Moreover, some non-neutralizing Abs targeting the non-RBD regions in the S protein may cause an antibody-dependent enhancement of SARS-CoV-2 infection, with consequent harmful immune response (71). Different from this study, Wan et al. showed that a MERS-CoV-specific neutralizing Mab targeting RBD mediated the entry of a MERS pseudovirus into Fc-expressing cells (72).

Importantly, some studies did not detect any cross-reactivity from other human coronaviruses (31, 35). Based on this observation, Amanat and collaborators excluded that the ADE from human coronaviruses might be the cause of the high pathogenicity of SARS-CoV-2 (35).

Further investigations are needed to understand the mechanism of ADE in facilitating viral infections and its putative role in COVID-19 onset and progression in order to address new viral vaccine design and antibody-based therapeutics.

## Discussions

The recent pandemic outbreak of SARS-CoV-2 virus and its rapid spread pose a urgent need for both diagnostic and therapeutic interventions to manage the containment measures of the infection and the outcome of the disease.

At the beginning of the epidemic outbreak, the Chinese government isolated and locked the Hubei province and as soon as the infection spread globally many countries implemented extraordinary measures to limit human-to-human transmission, especially that driven by asymptomatic people. Symptomatic people are testing for COVID-19 diagnosis; however, the molecular test based on the detection of the viral RNA that is currently used for the screening has some limitations. RT-PCR needs around 2–3 h to generate results, requires certified laboratories, expensive equipment, and often gives false negative results due to low viral load in the nasal and pharyngeal swabs. SARS-CoV-2 is a low respiratory tract-tropic virus, and sputum has a higher viral RNA positive rate than nasal swabs (73, 74). Moreover, the probability of a positive test seems to decrease with time since the onset of symptoms (74). Therefore, a huge number of symptomatic subjects might not be detected, improving the spread of the virus. Therefore, rapid and sensitive methods to screen the population are urgently needed. Serological tests might give a strong support to the diagnosis, complementing the molecular test, as several reports showed the presence of an antibody response in absence of detectable viral load. To date, none immunoassay has been reviewed and approved by FDA and the majority of the in-house assays require test in a statistical significant number of people to assess their performance.

In addition, serological tests are relevant to deeper characterize the SARS-CoV-2 specific antibody response. Differences in the profile of the antibody response across patients might reveal important aspects of the pathogenesis of COVID-19, explaining the great differences observed in the general population. Indeed, the correlation with disease severity and clinic characteristics is poorly understood. Old age and comorbidities seem to increase the risk of poor outcome of the disease; however, increasing cases of young people who experience severe illness, requiring hospitalization for assistance by mechanical ventilation may pose questions about the leading factors of disease progression.

Moreover, a deeper characterization of neutralizing antibodies might give insight on the potency and duration of the humoral immune response elicited by SARS-CoV-2. Indeed, researchers are trying to figure out whether patients can be re-infected by the virus after they recover from the primary infection. Some recovered COVID-19 patients from China, Sud Korea and Japan were tested positive for SARS-CoV-2 after discharge. However, the sera of convalescent patients appear useful to treat SARS-CoV-2 infected patients. The characterization of the humoral immune response of these patients will elucidate the mechanism of protection and will guide through the development of specific SARS-CoV-2 recombinant antibodies as prophylactic and therapeutic options to manage the disease. Some challenges are posed by the potential cross-reactivity with other human coronaviruses, due to their high homology at genetic level. The evidences related to this aspect are still controversial; however, SARS-CoV specific antibodies are undetectable in the sera of patients 6 years after infection. This observation excludes the presence of cross-reactivity in the sera of COVID-19 patients (75) and might make researchers confident about the specificity of these antibodies. Moreover, it would be interesting understanding whether the differences in the progression of the disease might be related to the level of the immune response. Certainly, a strong immune response leading to the recruitment and hyperactivation of immune cells ultimately triggers the cytokine storm that is an important cause of death in coronaviruses infection. Indeed, immune cells in the respiratory tract mediated the excessive and prolonged cytokine/chemokine response during the later stages of the infection of SARS-CoV and MERS-CoV, causing ARDS or multiple-organ dysfunction, which determined the poor outcome in patients. Therefore, together with the viral target it should be important taking into account the virus-mediated responses causing deleterious effects complicating the prognosis. In this light, blockade of cytokines and cytokine signaling pathways might represent useful therapeutic options for those patients undergoing cytokine storm. The CCR5 antagonist Leronlimab, a humanized monoclonal PRO 140 γ4-chain antibody (PRO 140), has already entered in a phase 2 randomized clinical trial for COVID-19 patients with mild-to-moderate symptoms (20). Tocilizumab, a recombinant humanized anti-human IL-6 receptor monoclonal antibody that specifically blocks the IL-6 receptor signaling pathway, is currently tested in a multicentre, randomized controlled trial in patients with COVID 19 pneumonia and elevated IL-6 in China (ChiCTR2000029765), showing promising results (76).

Not all the studies we reviewed here underwent the peer-reviewed process; therefore, they need to be confirmed. Further studies are rapidly needed to explore both the dynamics and the mechanisms of the humoral immune response in COVID-19 patients, in order to develop effective diagnostic and therapeutic options for the management of the disease.

## Author Contributions

GS wrote the manuscript. CP contributed to the revision of the literature. LL decided the structure and revised the manuscript.

## Conflict of Interest

The authors declare that the research was conducted in the absence of any commercial or financial relationships that could be construed as a potential conflict of interest.
